# Spatial analysis of care for patients undergoing dialysis therapy in the state of Minas Gerais, Brazil, between 2015 and 2019

**DOI:** 10.1590/1980-549720240002

**Published:** 2024-01-19

**Authors:** Claudio Vitorino Pereira, Isabel Cristina Gonçalves Leite, Mário Círio Nogueira, Gustavo Fernandes Ferreira

**Affiliations:** I Universidade Federal de Juiz de Fora, Department of Public Health – Juiz de Fora (MG), Brazil.; II Hospital Santa Casa de Misericórdia de Juiz de Fora, Department of Nephrology – Juiz de Fora (MG), Brazil.

**Keywords:** Renal dialysis, Healthcare disparities, Spatial analysis, Regional health planning, Diálise renal, Disparidades em assistência à saúde, Análise espacial, Regionalização da saúde

## Abstract

**Objective:**

To analyze the spatial flow of care for patients undergoing dialysis therapy in the health regions of the State of Minas Gerais.

**Methods:**

Ecological study whose population was patients undergoing dialysis therapy in public, philanthropic institutions or whose treatment was paid for by the Unified Health System in private clinics in partnership, in the State of Minas Gerais. Patients were grouped by health region of residence. The proportions of patients who underwent dialysis were calculated, as well as enrollment on the kidney transplant list in their own region of residence or outside it. Person correlations of these proportions with socioeconomic and care indicators of the health regions were estimated. Spatial exploratory techniques estimated general (Moran’s I) and local (LISA) spatial correlation coefficients.

**Results:**

Regions with higher GDP had a higher number of nephrologists and a higher proportion of registrations in the region of residence. A cluster of regions with low GDP was identified further to the northeast of the State (also with lower nephrologist ratio values), a cluster with a high proportion of those registered on the transplant list in the center of the State, and a cluster with a low proportion of dialysis in the same region of residence further southeast.

**Conclusion:**

Regional disparities were evident in relation to the proportion of patients registered on the waiting list for kidney transplantation, the proportion of patients undergoing dialysis in the same region of residence and the proportion of patients registered on the waiting list for kidney transplantation in the same region of residence. residence.

## RESUMO

**Objetivo:**

Analisar o fluxo espacial da assistência de pacientes em terapia dialítica nas regionais de saúde do estado de Minas Gerais.

**Métodos:**

Estudo ecológico que teve como população pacientes incidentes em terapia dialítica em instituições públicas, filantrópicas ou que tiveram seu tratamento custeado pelo Sistema Único de Saúde em clínicas privadas conveniadas, no estado de Minas Gerais. Os pacientes foram agregados por regional de saúde de residência. Foram calculadas as proporções de pacientes que fizeram diálise, bem como a inscrição na lista de transplante renal em sua própria região de residência ou fora dela. Estimadas as correlações de Person destas proporções com indicadores socioeconômicos e assistenciais das regionais de saúde. Técnicas exploratórias espaciais estimaram coeficientes de correlação espacial geral (I de Moran) e local (LISA).

**Resultados:**

Regiões com maior PIB apresentaram maior razão de nefrologistas e maior proporção de inscrições na própria região de residência. Identificou-se um cluster de regiões com PIB baixo mais ao nordeste do estado (também com valores mais baixos de razão de nefrologistas), um cluster de alta proporção de inscritos na lista de transplante no centro do estado, e um cluster de baixa proporção de diálise na mesma região de residência mais ao sudeste.

**Conclusão:**

Evidenciou-se disparidades regionais em relação à proporção de inscritos na lista de espera para o transplante renal, proporção de pacientes que realizavam diálise na mesma região de residência e proporção de pacientes inscritos na lista de espera para o transplante renal na mesma região de residência.

## INTRODUCTION

Regional inequities in access to renal replacement therapies and the growth in the number of patients on dialysis therapy have been identified as a global challenge to be overcome to improve the quality of treatment in nephrology^1^. The impacts of geographic heterogeneity on the availability of services, need for travel, socioeconomic factors, probability of inclusion of patients on the waiting list according to the dialysis center, and rate of kidney transplantation performed by region stand out^2^.

In Brazil, there was an increase of approximately 20.4% of the population undergoing dialysis treatment, in the period between 2015 and 2019^3^. In addition to the considerable increase in the percentage of patients on dialysis, the imbalance in the distribution of care services deserves special attention, as the Southeast region concentrates 47% of active dialysis centers in the country, and approximately 48.3% of medical professionals specialized in nephrology^3^.

The state of Minas Gerais (MG) is marked by socioeconomic inequities between its regions, with areas of high economic development and locations marked by extreme poverty^4^. According to data from the latest Brazilian Dialysis Census, it is estimated that MG has 20,314 patients undergoing dialysis treatment, with a prevalence of 949 per million people^3^. In relation to the waiting list, the state had, at the end of 2021, 2,923 patients with an active registration on the waiting list for kidney transplantation, a value corresponding to 14.3% of the population on dialysis^5^.

With a view to reorganizing and improving conditions of access to health services in MG, the Regionalization Master Plan established the Regional Health Superintendences (*Superintendências Regionais de Saúde* – SRS) and the Regional Health Managements (*Gerências Regionais de Saúde* – GRS) as administrative divisions to support management of the Brazilian Unified Health System (*Sistema Único de Saúde* – SUS), due to the territorial extension and number of municipalities in the state^6^. Regarding the structure of the State Transplant System, the state has the State Transplant Center and seven Organ Procurement Organizations (OPOs), whose geographic distribution was based on the division of GRS and SRS^7^.

The equitable distribution of specialized services in highly complex nephrology care is paramount to minimize the impacts caused by renal replacement therapies. Patients who require long commuting to the dialysis center have shown a decline in health indicators^8^.

As it is a chronic, costly condition with repercussions on the daily lives of people who require dialysis therapy, as well as their family caregivers, it is essential to analyze the current scenario of individuals’ access to dialysis treatment.

The present study aimed to analyze the distribution and spatial flow of care for patients undergoing dialysis therapy in the health regions MG and the associated contextual factors.

## METHODS

This is an ecological study on patients undergoing dialysis therapy from 01/01/2015 to 12/31/2019, in public, philanthropic institutions or whose treatment was paid for by SUS in private affiliated clinics in MG.

The state of MG is located in the Southeast region of Brazil, has the fourth largest territorial extension among Brazilian states, and the second largest population. It stands out for being the Brazilian state with the largest number of municipalities, with 853 cities. However, 55.8% of them have up to 10,000 inhabitants, and only 32 have populations above 100,000 inhabitants^6^.

Data sources were: status on the waiting list for kidney transplants registered in the National Transplant System (*Sistema Nacional de Transplante* – SNT) and for Authorization for High Cost Procedures (*Autorização para Procedimento de Alto Custo* – APAC), both provided by SNT in Excel spreadsheets, with a unique identification of patients, which allowed their deterministic relationship and the creation of a single bank based on the integration of information about dialysis procedures and registration on the waiting list for each patient.

Records that were duplicates or that had a place of residence outside MG were excluded. The sample included records of patients aged 18 years old or older, undergoing dialysis between January 2015 and December 2019 and undergoing chronic dialysis treatment, characterized by at least 90 days on dialysis. Initial database contained 33,915 registered patients; Of these, the following were excluded: 4,031 patients who started dialysis before 2015, 610 who lived outside MG, 288 due to being under 18 years old, and 5,689 because they had been on dialysis for less than 90 days. Therefore, study population consisted of 23,297 patients.

Patients were grouped by health region of residence (SRS or GRS; n=28). For each patient, the health region where they started dialysis treatment and the region where they were registered on the kidney transplant waiting list, if registered, were identified.

For each health region, the proportions of patients who underwent dialysis in their own region of residence (called internal dialysis flow) and the proportion in another region of residence (external flow) were calculated. The same analysis was carried out for registration on the transplant list, identifying internal and external flows.

The proportions of registration on the transplant list and internal flows were calculated and presented on thematic maps, with quintiles of their distribution. To represent patient care flows, maps were created with arrows between the centroid of the region of residence and the centroid of the region of care, with the thickness of the arrow proportional to the number of patients.

The association (by Person correlation) of these proportions with socioeconomic and care indicators of the health regions was evaluated, based on data tabulated on the page of the SUS Information Technology Department (*Departamento de Informática do SUS* – DATASUS). Considering a conceptual model that proposes that the socioeconomic situation of the region is a determinant of the available care resources, and these in turn condition access to health services and actions, the following indicators were selected: GDP *per capita* (mean from 2010 to 2013), Family Health Strategy (FHS) coverage in 2015 and ratio of nephrologists per 10,000 inhabitants (mean from 2015 to 2019).

Finally, as spatial exploratory techniques, the general (Moran’s I) and local (LISA) spatial correlation coefficients were estimated, the latter presented as a map of significant clusters with the identification of discrepant regions in relation to their surroundings, with the clusters LOW/HIGH or HIGH/LOW. The unification of spreadsheets, statistical analyses and map illustrations were carried out in R software, v. 4.1.0.

The present study was approved by the Research Ethics Committee of the University Hospital of Universidade Federal de Juiz de Fora (MG), process number 1.709.611.

## RESULTS

23,297 records of patients undergoing dialysis therapy in MG were analyzed. In the 28 health regions of MG, GDP *per capita* ranged from R$ 5,552 to R$ 33,995, the coverage of FHS from 17.50 to 84.90%, and the ratio of nephrologists from 0 to 0.44 per 10,000 inhabitants, which shows a wide difference in socioeconomic and care indicators (Table 1).

**Table 1:** Sociodemographic and care indicators of health regions in the state of Minas Gerais, 2015–2019.

Characteristic	Mean	SD	Min	Q1	Q2	Q3	Max
GDP *per capita*	17,262.29	7,509.07	5,552.00	10,996.00	17,069.00	20,287.00	33,995.00
Cov. FHS	57.35	17.15	17.50	48.28	59.55	72.45	84.90
Nephro *per capita*	0.18	0.10	0.00	0.12	0.16	0.21	0.44
Proportion of registrations	12.73	5.92	3.90	7.20	12.75	15.73	23.40
Proportion of domestic dialysis	88.84	16.70	32.30	89.75	95.80	97.70	99.90
Proportion of domestic registration	25.63	41.51	0.00	0.00	0.00	56.80	100.00

SD: standard deviation; Min: minimum value; Q1: first quartile; Q2: second quartile; Q3: third quartile; Max: maximum value. GDP: gross domestic product. Cov. FHS: coverage of the Family Health Strategy. Nephro per capita: ratio of nephrologists per 10,000 inhabitants. Proportion of registrations: on the transplant list. Proportion of domestic dialysis: proportion of patients who undergo dialysis in their own region of residence. Proportion of domestic registration: proportion of patients who were registered on the transplant list in their region of residence.

Source: DATASUS

There was also a wide variation in indicators related to chronic kidney disease (CKD), such as the proportion of those registered on the kidney transplant waiting list, from 3.90 to 23.40%; the proportion of patients undergoing dialysis in the same region of residence ranged from 32.30 to 99.90%; and the proportion of patients registered on the kidney transplant waiting list in the same region of residence ranged from 0 to 100% (Table 1). Data related to sociodemographic and care indicators from the health regions in MG are presented in Table 1.

When observing Table 2, it is clear that the only variables with a significant correlation between them were: GDP *per capita* with ratio of nephrologists (r=0.504) and with proportion of registered in the region of residence (r=0.460), and ratio of nephrologists with proportion of enrollees in the region of residence (r=0.654). Only one variable had a significant spatial correlation, the proportion of registration in the region of residence, with Moran’s I of 0.25 (p=0.008) (Table 2).

**Table 2:** Pearson correlation coefficient between care variables.

Characteristics	Cov. FHS 2015	Nephrol. pc.	Prop. reg.	Prop. Domestic dialysis	Prop. Domestic reg.
GDP pc	- 0.218	0.504*	-0.053	-0.076	0.460*
Cov. FHS 2015	–	0.134	-0.033	-0.090	0.016
Nephrol. Pc	–	–	0.066	0.199	0.654*
Prop. Reg.	–	–	–	-0.274	0.189
Prop. Domestic dialysis	–	–	–	–	0.235

GDP pc: Gross Domestic Product *per capita*; Cov. FHS 2015: Coverage of the Family Health Strategy 2015; Nephrol pc: Nephrologists *per capita*; Prop. Reg.: Proportion of those registered on the kidney transplant waiting list; Prop. Domestic dialysis: Proportion of dialysis in the same health region as the residence; Prop. Domestic reg..: Proportion of those registered on the waiting list for kidney transplantation in the same region of residence; *p value < 0.05.

**Figure 1.**
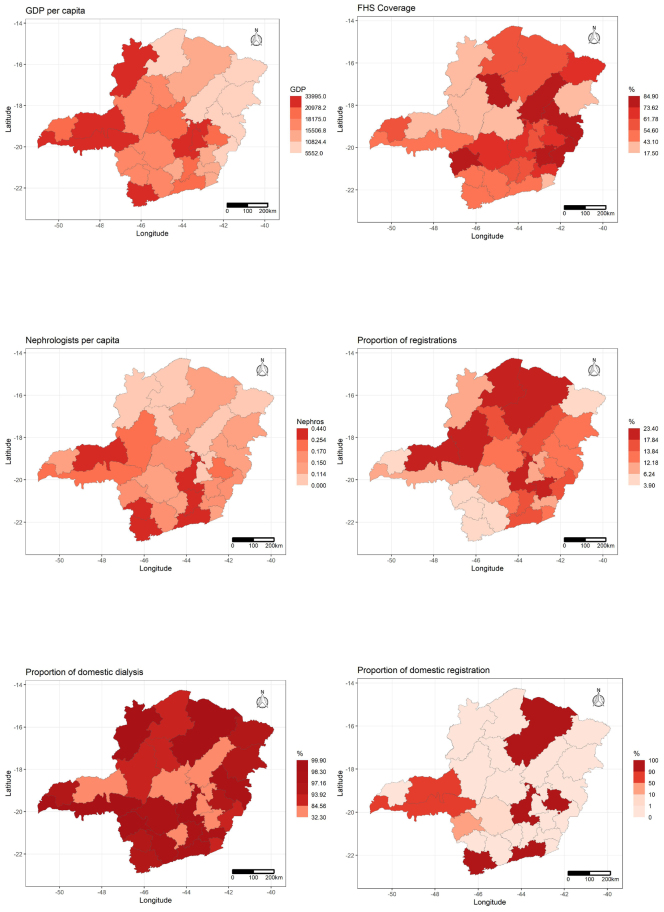
Spatial distribution by quintiles of socioeconomic and care variables by health regions in the state of Minas Gerais, 2015–2019.

**Figure 2.**
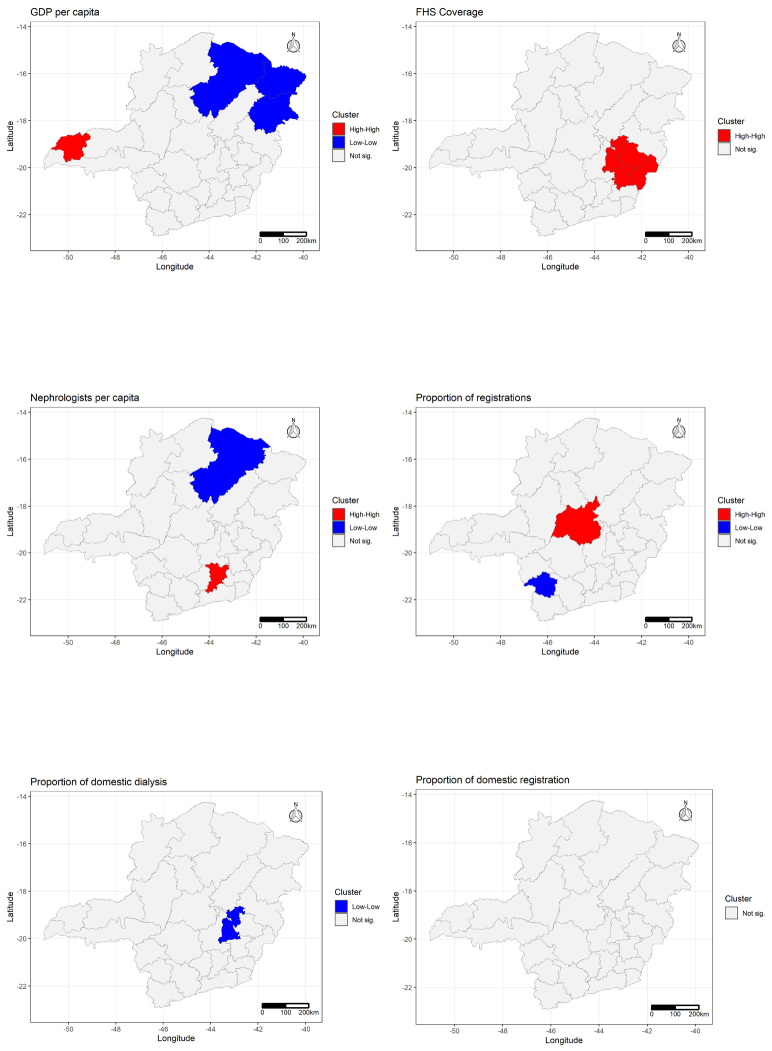
Map of local spatial autocorrelation clusters (LISA map) of socioeconomic and care variables by health regions in the state of Minas Gerais, 2015–2019.

The maps with the distributions of variables by quintiles show that the regions with the highest GDP are also those with the highest number of nephrologists and the highest proportion of registrations in the region of residence. Analyzing the local spatial correlation map (LISA), a cluster of regions with low GDP is identified further to the northeast of MG (which also has low nephrologist ratio values), a cluster of regions with high FHS coverage in the Central East of the state, and a cluster with a high proportion of those registered on the transplant list in the center of the state, with a cluster with a low proportion of dialysis in the same region of residence, a little to the South and to the East (Figures 1 and 2).

**Figure 3.**
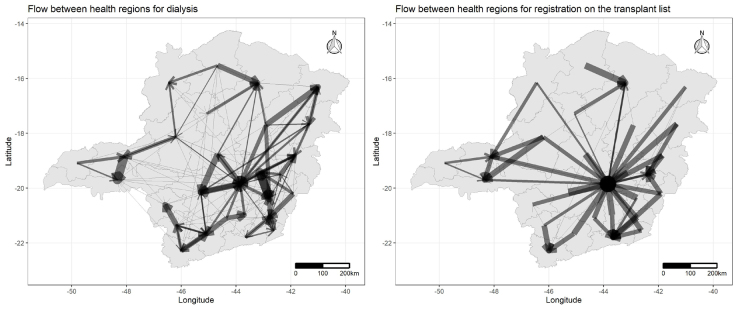
Care flows for dialysis and registration on the waiting list for kidney transplantation in health regions in the state of Minas Gerais, 2015–2019.

Figure 3 shows the care flows in the health regions of the state of MG for dialysis and registration on the waiting list for kidney transplantation. Through the analysis of the maps, a variety of external flows of dialysis patients to neighboring regions can be identified, while in the case of registration on the kidney transplant list there are greater displacements and the predominance of external flows is to the Belo Horizonte region, state capital (Figure 3).

## DISCUSSION

It is noteworthy that the scenario experienced by people with CKD is characterized by strong sociodemographic, ethnic, and geographic disparities throughout the world^9,10^, which are reflected in the distribution of health services intended for patients undergoing renal replacement therapy (RRT), as well as access to existing services^11^.

The present study highlighted socioeconomic and healthcare differences represented by GDP *per capita*, FHS coverage and availability of nephrologists in the state’s health regions. The indicators proportion of those registered on the transplant list, proportion of patients undergoing dialysis in the same region of residence, and proportion of patients registered on the waiting list for kidney transplantation in the same region of residence, which are related to the quality of care for patients in RRT also varied widely across different health regions. Sociodemographic inequities, local specificities and unequal distribution of resources impose difficulties in accessing the various health services in MG^6^, including care aimed at patients undergoing dialysis therapy.

The complex and expensive nature of RRT is closely related to economic status, with the best care indicators and greater supply of resources for nephrological treatment available in locations with higher GDP^12^. This research found a positive correlation between GDP *per capita* with the number of nephrologists and the proportion of enrollees in the region of residence. There was also a positive association between the ratio of nephrologists and the proportion of enrollees in the region of residence.

Economic-structural differences between locations have an impact on all stages of care to individuals undergoing dialysis therapy and the possibilities of obtaining a kidney transplant^13^. In Brazil, analysis of data related to high complexity care in nephrology demonstrates that the substantial increase in patients on dialysis therapy has not been accompanied by the growth of patients on the waiting list for kidney transplantation and the number of kidney transplants performed^5^. As a result, there is increasing difficulty in accessing specialized care. Low-income countries have less availability of Transplant Centers and a low supply of nephrologists^12^. Greater availability of nephrologists may be associated with an increased likelihood of enrollment on the kidney transplant waiting list, just as low socioeconomic status is negatively associated with enrollment^14^.

The analysis of the local spatial correlation map favors the analysis of the results of economic and structural disparities in MG, making it possible to identify a cluster of regions with low GDP further to the northeast of MG, where a low ratio of nephrologists was also found. Such findings reinforce concerns about the structuring of the care network in the northeast macro-region, previously highlighted in other studies, with evidence of organizational failure of the network to provide therapeutic resources, in addition to high dependence on other macro-regions for care to medium and large burns^15^. Another research demonstrated that the northeast macro-region has the highest mean traveled distance to carry out hemodialysis sessions in the state^16^.

Organizational relevance of the state capital and metropolitan region can also be highlighted, as there is a cluster of high FHS coverage in the Central East of the state, as well as a high proportion of those registered on the transplant list in the center of the state. FHS is seen as an important tool for managing the care of CKD patients, with prevention, disease screening, and timely referral to a specialized nephrology service as highlights, which could result in a better clinical condition when starting RRT^17^. The existence of resources can be evidenced when analyzing the distance traveled for care, in the case of medium and large burns^15^ or even patients on hemodialysis therapy^16^, in which the Central macro-region had one of the lowest travel needs.

Geographical disparities, lack of resources and/or vacancies for care, or even the search for higher quality care, can have an impact on the migration of patients between regions^16^. The Belo Horizonte region had the highest flow of other regions to register on the kidney transplant waiting list, a fact that may be associated with its protagonism due to the high number of kidney transplants performed annually, as well as the logistics of the care network with the largest variety of services. Studies highlight that geographic remoteness from large centers, locations with low socioeconomic indicators and insufficient health-related structure negatively impacts the enrollment of patients on the waiting list, as well as the number of kidney transplants performed^14,18^.

The present study enabled a new analysis of the situation of High Complexity nephrology treatment in the health regions of MG, through data from all individuals who underwent dialysis therapy in the aforementioned period. Limitations of the present study are related to the secondary nature of the data extracted from APAC, in which information that was not mandatory for financial transfer was sometimes incomplete.

The results of the present study showed regional disparities in relation to the proportion of patients registered on the waiting list for kidney transplantation, the proportion of patients undergoing dialysis in the same region of residence and the proportion of patients registered on the waiting list for kidney transplantation in the same region of residence. Regions with the highest GDP are also those with the highest number of nephrologists and the highest proportion of registrations in the region of residence.

It is a priority to reformulate care for individuals with CKD undergoing dialysis treatment, with a view to optimizing access to services, increasing the quality of care offered, and ensuring equity at all stages, that is, so that there are patients in better clinical conditions, along with greater knowledge about the pathology and treatment, in addition to an increase in the referral of patients on the waiting list and expansion of kidney transplant surgeries.

It is expected that the present study will stimulate debates on the need to optimize the care structure in nephrology in the state of MG, as well as awareness about the geographic and socioeconomic barriers that affect access to dialysis therapies and kidney transplantation. Therefore, it is necessary to establish and disseminate care indicators for the state’s health regions, so that the quality of locations with satisfactory results is maintained and, in deficient regions, there is joint intervention by Public Authorities, Dialysis Centers, and Transplantation Centers.

## References

[1] Paul S, Plantinga LC, Pastan SO, Gander JC, Mohan S, Patzer RE (2018). Standardized transplantation referral ratio to assess performance of transplant referral among dialysis facilities. Clin J Am Soc Nephrol.

[2] Ross-Driscoll K, Axelrod D, Lynch R, Patzer RE (2020). Using geographic catchment areas to measure population-based access to kidney transplant in the United States. Transplantation.

[3] Sociedade Brasileira de Nefrologia (2021). Censo brasileiro de diálise 2021 [Internet].

[4] Pereira NJ, Souza KR (2018). Pobreza no estado de Minas Gerais: uma análise da região Norte. Revista Iniciativa Econômica.

[5] Associação Brasileira de Transplante de Órgãos (2021). Dimensionamento dos transplantes no Brasil e em cada estado 2014–2021 [Internet].

[6] Governo do Estado de Minas Gerais (2020). Secretaria de Estado de Saúde de Minas Gerais.. Plano estadual de saúde de Minas Gerais: 2020–2023 [Internet].

[7] Governo do Estado de Minas Gerais (2019). Secretaria de Estado de Saúde. Fundação Hospitalar do Estado de Minas Gerais. Central Estadual de Transplantes. Plano estadual de doação e transplantes de órgãos e tecidos 2019–2023 [Internet].

[8] Pereira E, Santos MA, Carvalho M (2021). Route of chronic kidney patients foreigners in the search for health care in a border area. Rev Bras Enferm.

[9] Murray R, Zimmerman T, Agarwal A, Palevsky PM, Quaggin S, Rosas SE (2021). Kidney-related research in the United States: a position statement from the National Kidney Foundation and the American Society of Nephrology. Am J Kidney Dis.

[10] Châtelet V, Lobbedez T, Harambat J, Bayat-Makoei S, Glowacki F, Vigneau C (2018). Précarité et greffe rénale: pourquoi et comment estimer son effet sur la santé des populations?. Nephrol Ther.

[11] Kiani B, Bagheri N, Tara A, Hoseini B, Hashtarkhani S, Tara M (2018). Comparing potential spatial access with self-reported travel times and cost analysis to haemodialysis facilities in North-Eastern Iran. Geospat Health.

[12] Crews DC, Bello AK, Saadi G (2019). Carga, acceso y disparidades en enfermedad renal. Arch Argent Pediatría.

[13] Wesselman H, Ford CG, Leyva Y, Li X, Chang CCH, Dew MA (2021). Social determinants of health and race disparities in kidney transplant. Clin J Am Soc Nephrol.

[14] Pruthi R, Robb ML, Oniscu GC, Tomson C, Bradley A, Forsythe JL (2020). Inequity in access to transplantation in the United Kingdom. Clin J Am Soc Nephrol.

[15] Souza MT, Nogueira MC, Campos EMS (2018). Fluxos assistenciais de médios e grandes queimados nas regiões e redes de atenção à saúde de Minas Gerais. Cad Saúde Colet.

[16] Pereira CV, Leite ICG (2019). Qualidade de vida relacionada à saúde de pacientes em terapêutica hemodialítica. Acta Paul Enferm.

[17] Paula PHA, Santos PR, Salles LD, Dias MSA, Pinheiro PNC, Costa MIF (2020). Assistência ao paciente renal antes do início da hemodiálise: estudo retrospectivo. Cienc Cuid Saúde.

[18] Thurlow JS, Joshi M, Yan G, Norris KC, Agodoa LY, Yuan CM (2021). Global epidemiology of end-stage kidney disease and disparities in kidney replacement therapy. Am J Nephrol.

